# Giant Spigelian Hernia presenting as small bowel obstruction: Case report and review of literature

**DOI:** 10.1016/j.ijscr.2019.09.026

**Published:** 2019-09-24

**Authors:** Marino Di Furia, Lucia Romano, Andrea Salvatorelli, Denise Brandolin, Gianni Lazzarin, Mario Schietroma, Francesco Carlei, Antonio Giuliani

**Affiliations:** Department of General Surgery, Hospital San Salvatore L’Aquila, University of L’Aquila, Italy

**Keywords:** Spigelian Hernia, Small bowel obstruction, Mesh repair

## Abstract

**Introduction:**

Spigelian Hernia is an uncommon pathology of abdominal wall (0.12–2.4%), usually small sized and with vague symptoms. It rarely presents as Small Bowel Obstruction or reaches dimensions that becomes clinically remarkable.

**Presentation of case:**

84-year-old woman entered our Surgical Department for Small Bowel Obstruction due to a giant (8 × 7 cm) abdominal wall hernia, which was intraoperatively identified as Spigelian Hernia. We performed a minilaparotomy with reduction of viable small bowel and preperitoneal positioning of polypropilene mesh. Postoperative course was uneventfull.

**Discussion:**

Due to its small dimensions and infrequence, the diagnosis could be challenging even if the patient undergoes a CT scan. The presentation with clear signs of small bowel obstruction associated with a large abdominal hernia is rare and suggests a urgent surgical approach with mesh repair to avoid recurrences.

**Conclusion:**

Even if rarely symptomatic, the Spigelian Hernia is an entity to consider in the differential diagnosis of small bowel obstruction in a virgin abdomen. Preoperative diagnosis, when available, is mandatory to guide a correct surgical approach.

## Highlights

• Spigelian Hernia is a rare abdominal wall hernia.
• Urgent presentation with small bowel obstruction is uncommon.
• Laparoscopy could be an option in elective surgery but in emergent setting laparotomy is required.
• Mesh repair is mandatory to avoid recurrence.

## 1 Introduction

Spigelian Hernia (SH) arises from a defect on the aponeurotic area firstly described by Adriaan van der Spieghel in 1645 and it is located between the lateral edge of the rectus abdominis muscle medially and the semilunar line laterally [1].

It rarely shows as a visible abdominal wall hernia because it tends to be intraparietal (located behind the external oblique aponeurosis) and rarely becomes bigger than 2 cm [2]. It can enlarge its dimensions after damage to a weakened EO aponeurosis, protruding the hernial sac in the subcutaneous tissue.

SH represents from 0.12% to 2.4% of all abdominal wall hernias and, like other type of hernias, is usually associated to risk factors such as obesity, age, multiparity, collagen disorders and chronic obstructive pulmonary disease [3].

The work has been reported in line with the SCARE criteria [4].

## 2 Presentation of case

A 84-year-old woman presented to out Emergency Department with a 36-h history of abdominal pain, nausea and bilioeneteric vomit. Her patency was absent from 3 days. She referred the appearance of a bulging mass in the left lower quadrant of the abdomen,

Her medical history was dominated by COPD, morbid obesity and cardiologic arrhythmia (treated with DOAC).

Blood exams revealed mild leukocytosis (WBC 12.34 × 109/L, increase in CRP and severe acute kidney injury.

**Fig. 1:**
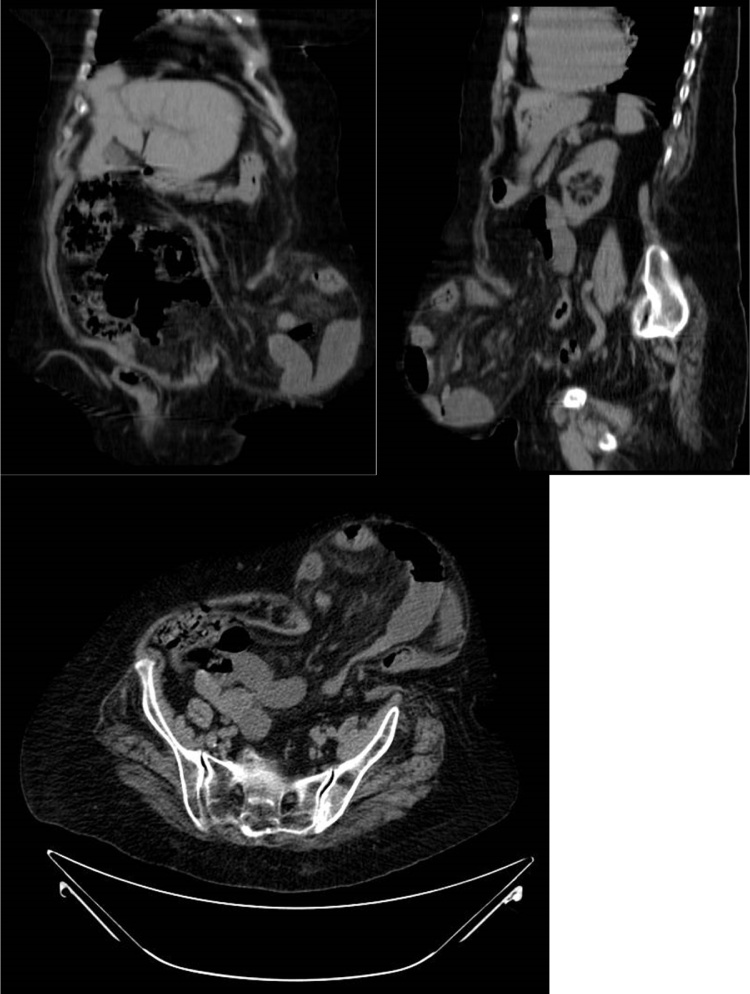
CT scan reveal several loops of small bowel introducing into a large defect (3 × 3 cm) on the left lateral abdominal wall, at the level of the iliac crest, and causing a SBO of the upper part.

Physical examination showed a distended abdomen and a bulging mass (8 × 7 cm) located in the left lower quadrant with tenderness and impossibility to obtain manual reduction. Auscultation revealed tympanic movement of bowel (Fig. 1).

**Fig. 2:**
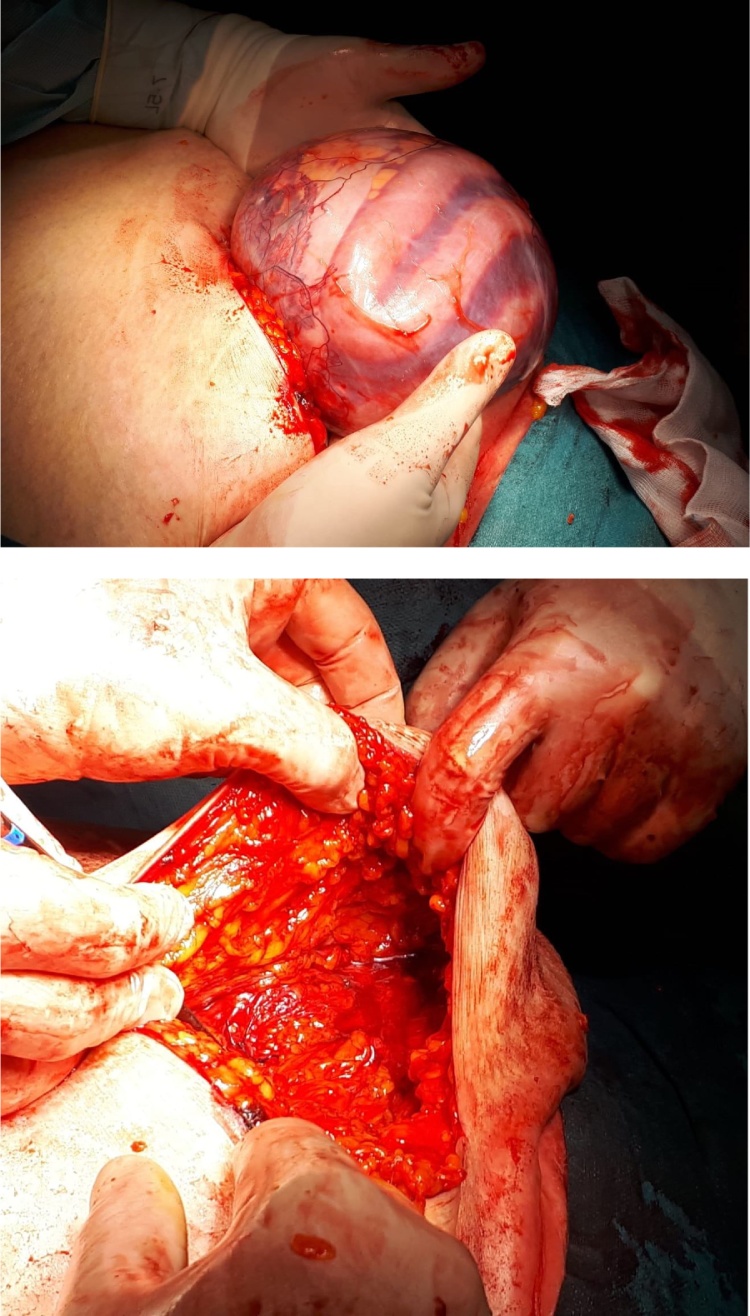
Intraoperative view of the hernia sac including several viable small loops and fluid (upper part) and of the abdominal wall showing the defect (Spigelian Hernia).

After placement of a nasogastric tube, the patient underwent an abdominal CT scan which revealed several loops of small bowel introducing into a large defect (3 × 3 cm) on the left lateral abdominal wall, at the level of the iliac crest, and causing a SBO of the upper part (Fig. 2).

Initial fluid resuscitation was performed and the patient was scheduled for urgent surgery.

Laparoscopic approach was not proposed because of the comorbidities and the high abdominal distension.

Minilaparotomy (5 cm) was performed under the umbilicus and identified the hernia sac, which was easily isolated from subcutaneous tissues and the muscolofascial plane. Opening the hernia sac, a small amount of clear fluid went out along with 60 cm of ileal loops which was viable and needed no resection (Fig. 2). The small bowel was reduced into the abdominal cavity and showed a 3 × 3 cm defect on the fascial plane. We chose to adopt a preperitoneal repair, closing the sac and positioning a 6.4 cm Ventralex™ hernia mesh into this space. Surgical procedure ended with the positioning of a subcutaneous drain and suture of the wound.

Postoperative was uneventfull; patency was obtained on 2nd postop day and the patient was discharged on 3rd with a semiliquid diet.

## 3 Discussion

Even if it is a rare hernia (from 0.12 to 2.4% of all hernias), incarceration is possible in a relevant percentage of case (17–24%) [2].

The most difficult part in the emergency setting is the diagnostic part: clinical history and examination are usually little helpful because of SH may cause vague abdominal pain which is often referred to other causes and, moreover, its intraparietal location makes SH not palpable from the clinician in almost 36% of cases [6].

Clinical suspect can be reinforced from a persistent abdominal pain and tenderness in the Spigelian point, but a correct diagnosis is impossible without imaging: US is a first-line useful method, especially in emergency settings, but if available the gold standard for diagnosis is still represented by CT scan, even if it has up to 32% of false negative [5,7,8].

Certainly, clinical presentation of SBO associated with bulging mass in the abdominal wall is very suggestive of obstructed hernia, but the definitive diagnosis of SH is made intraoperatively in almost all cases.

We think our case is very interesting because it shows a giant Spigelian hernia (according the most common classification, a giant hernia is considered if the dimensions are major than 5 cm) with an obstructive presentation, which is not so common among these rare abdominal wall herniations (described in few case reports).

Surgical approach is urgent in the majority of cases and requires laparotomy (or at least, abdominal incision) due to the distended bowel loops for the SBO; in our cases, the poor clinical status of this elderly patient, the high risk of having a poor tissue regenerative power, associated with the non-resolutive CT scan forced us to perform a minilaparotomy to enlarge the surgical field [9,10].

Laparoscopy could be a good approach especially in elective surgery with both intra and extraperitoneal mesh placement that seem equivalent [11] but it rarely can be used in urgent surgery due to abdominal distension consequent to SBO.

Therefore, we suggest not to perform incision directly above the mass unless the preoperative diagnosis is certain or at least very suggestive of SH.

Mesh repair is the preferred option also in urgent surgical procedures due to the little incidence of infection and the rare need for intestinal resection; recurrence rate is low (0–8%) [12,13].

## 4 Conclusion

Among all the possibilities in differential diagnosis for SBO, Spigelian Hernia still has an important place. Preoperative diagnosis by CT scan could be essential to guide the surgeon to a mininvasive approach, even if in most cases urgent laparotomic surgery is needed due to abdominal distension.

## Funding

No source of funding.

## Ethical approval

This study is exempt form ethical approval.

## Consent

Consent was obtained from the patient.

## Author’s contribution

Brandolin Denise and Giuliani Antonio performed the surgical procedure.

Lazzarin Gianni and Romano Lucia collected data and pictures from surgery.

Di Furia Marino and Salvatorelli Andrea proposed the study and wrote the paper.

Schietroma Mario and Carlei Francesco supervised the paper and controlled all the analysis of results, including language.

## Registration of research studies

Not needed.

## Guarantor

Dr. Di Furia Marino.

## Provenance and peer review

Not commissioned, externally peer-reviewed.

## Declaration of Competing Interest

No conflict of interest.
